# Antimicrobial Activity Profiles and Potential Antimicrobial Regimens against Carbapenem-Resistant Enterobacterales Isolated from Multi-Centers in Western Thailand

**DOI:** 10.3390/antibiotics11030355

**Published:** 2022-03-07

**Authors:** Parnrada Nulsopapon, Manat Pongchaidecha, Worapong Nasomsong, Pitimon Polwichai, Sirilada Suphankong, Pantip Sirichote, Siriwan Chaisomboonpan, Wichai Santimaleeworagun

**Affiliations:** 1 Department of Pharmacy, Faculty of Pharmacy, Silpakorn University, Nakhon Pathom 73000, Thailand; aom.olivegreen1812@gmail.com (P.N.); pongchaidecha_m@su.ac.th (M.P.); 2 Pharmaceutical Initiative for Resistant Bacteria and Infectious Diseases Working Group [PIRBIG], Faculty of Pharmacy, Silpakorn University, Nakhon Pathom 73000, Thailand; 3 Department of Internal Medicine, Division of Infectious Diseases, Phramongkutklao Hospital and College of Medicine, Bangkok 10400, Thailand; nasomsong.w@gmail.com; 4 The Regional Medical Sciences Center 5, Samutsongkhram 75000, Thailand; pitimon.p@dmsc.mail.go.th (P.P.); sirilada.s@dmsc.mail.go.th (S.S.); pantip.s@dmsc.mail.go.th (P.S.); siriwan.c@dmsc.mail.go.th (S.C.)

**Keywords:** CRE, Enterobacteriaceae, *mcr-1*, NDM, OXA-48

## Abstract

The spread of carbapenem-resistant Enterobacterales (CRE) constitutes a global health burden. Antimicrobial susceptibility and types of carbapenemase differ by geographic region. This study aimed to (1) examine the minimum inhibitory concentrations (MICs) and antibiotic resistance genes and (2) investigate antibiotic dosing regimens against CRE using Monte Carlo simulation. Clinical carbapenem-resistant *Klebsiella pneumoniae* (CRKP), *Escherichia coli* (CREC), and *Enterobacter cloacae* (CREclo) isolates were collected from various hospitals in western Thailand. Broth microdilution was performed, and the types of carbapenemase and *mcr-1* genes were detected using polymerase chain reaction (PCR). Monte Carlo simulation was used to establish optimal antimicrobial dosing regimens meeting the criterion of a cumulative fraction of response (CFR) >90%. A total of 150 CRE isolates from 12 hospitals were included. The proportion of CRKP (76%) was greater than that of CREC (22%) and CREclo (2%). Regional hospitals reported higher rates of resistance than general hospitals. Most isolates were resistant to aztreonam and ceftazidime/avibactam, whereas they were highly susceptible to aminoglycosides. Most carbapenemases were NDM (47.33%), OXA-48 (43.33%) and NDM plus OXA-48 (6.67%); five OXA-48 positive isolates carried *mcr-1* genes. Currently, high-dose tigecycline is the only optimal regimen against CRE isolates. Further extensive research on antibiotic synergism or new antibiotics should be conducted.

## 1. Introduction

The development of antibiotic resistance is rapidly changing and represents a serious global health challenge. Carbapenem-resistant Enterobacterales (CRE), especially carbapenem-resistant *Klebsiella pneumoniae* (CRKP), *Escherichia coli* (CREC) and *Enterobacter cloacae* (CREclo), represent critical Gram-negative bacteria with resistance to carbapenems and multiple antibiotics. In 2017, the World Health Organization (WHO) placed the pathogens in the highest priority list [1]. Invasive CRE infections have been associated with mortality rates of 40 to 50% [2]. The risk of death among patients infected with CRE is 3 fold higher than among patients infected with carbapenem-susceptible Enterobacterales [3]. Bartsch et al. estimated the economic burden from CRE epidemiology and reported an increase in incidence from 2.93 to 15 per 100,000, and medical costs increased from USD 275 to 1406 million [4]. In Thailand, an increase has been observed in the burden of CRE. The prevalence of CRE identified from clinical isolates of Enterobacterales rose from 1.1 to 13.2% from 2010 to 2020 [5]. Phodha T et al. estimated that the excess treatment costs for multi-drug resistance (MDR) *K. pneumoniae* and MDR *E. coli* were USD 14,921 and USD 13,502, respectively [6]. Among CRE, the most common species are *K. pneumoniae*, *E. cloacae* and *E. coli* [7], whereas the most common CRE species in Thailand are *K. pneumoniae* and *E. coli* [5].

CRE can cause healthcare-associated infections (HCAIs), such as bloodstream infections (BSI), pneumonia, complicated intraabdominal infections (cIAIs) and complicated urinary tract infections (cUTIs) [8]. The main mechanisms of resistance are classified in two groups: carbapenemase production (CP-CRE) and non-carbapenemase production (non-CP-CRE). Focusing on CP-CRE, the Ambler classification system is commonly used to classify β-lactamases. Serine is required as an active site residue in Ambler classes A and D, whereas zinc is an essential cofactor for breaking the β-lactam ring in Ambler class B. Common types of carbapenemases in Ambler classes A, B and D consist of *K. pneumoniae* carbapenemase (KPC), New Delhi metallo-β-lactamase (NDM) and oxacillinase-48 (OXA-48) [9]. In 2017, a study showed that CP-CRE may be more virulent than non-CP-CRE because a large proportion of meropenem minimum inhibitory concentrations (MICs) were ≥4 µg/mL, and most types of CP-CRE isolates in the study were *bla_KPCs_* (92%) [10]. Furthermore, Fattouh R et al. showed data on associations between MIC and types of carbapenemase genes. Overall, all NDM-positive isolates had meropenem MICs ≥ 2 µg/mL, whereas OXA-48- and KPC-positive isolates were relatively distributed across a meropenem MIC range from ≤0.12 to ≥16 µg/mL [11]. It seems that the type of carbapenemase gene may affect MIC levels.

Carbapenemases have different geographic distributions. The most commonly occurring KPC-producing CRE (class A) has been reported in the US, while metallo-β-lactamase (MBL)-producing CRE (class B) is mostly found in the Indian subcontinent, Romania, Denmark, Spain and Hungary. Furthermore, OXA-48-like-producing CRE (class D) is common in Turkey and surrounding countries [12].

In Thailand, several studies showed that the molecular characteristics of carbapenemase are diverse in each setting. Rimrang B et al. collected Enterobacteriaceae from a university hospital in the northeast region between 2010 and 2011 and could detect only four IMP-14a-producing and six NDM-producing isolates [13]. In the western region, Preechachuawong P et al. collected clinical Enterobacteriaceae isolates from a general hospital in 2014. They found only NDM-producing isolates from one *K. pneumoniae* isolate [14]. In the central region, Netikul T et al. obtained 181 clinical CRE isolates from a large university hospital from 2009 to 2011. The reported CP-CRE types included three KPC-13-producing and four IMP-14a-producing isolates [15]. From 2012 to 2016, Laolerd W et al. collected 223 CRE isolates harboring carbapenemase from a tertiary hospital, and the following carbapenemase enzymes were found: NDM (46.64%), NDM plus OXA-48-like (25.11%) and OXA-48-like (25.11%), IMP (3.14%), VIM (0%), and KPC (0%) [16]. Furthermore, Nasomsong W et al. collected 49 CRE isolates from a university hospital in 2020, and most expressed carbapenemases, including OXA-48-like (53.11%), NDM plus OXA-48-like (42.9%) and NDM (2%) [17]. Diverse carbapenemase enzymes that cause differences in MIC levels may contribute to different antibiotic options and antibiotic dosing regimens to treat CRE.

Currently, antibiotic options to CRE treat infections remain extremely limited, with colistin, tigecycline and aminoglycosides constituting the mainstay of treatment. Recently, new antibiotic options, namely, aztreonam and ceftazidime/avibactam, have been reappraised or developed as new generation antibiotics. The addition of avibactam to ceftazidime increases the spectrum of activity against CRE species that produce ESBLs and serine carbapenemases (KPC; OXA-48 and its derivatives) but is not active against class B carbapenemases. Furthermore, aztreonam exhibits specific activity against MBL producers [18]. A clinical study showed that the 30-day adjusted mortality of patients with KPC-producing isolates was lower following treatment with ceftazidime/avibactam compared with colistin (ceftazidime/avibactam vs. colistin = 9% vs. 32%; *p* = 0.001) [19]. In 2020, the Infectious Diseases Society of America recommended ceftazidime/avibactam and ceftazidime/avibactam plus aztreonam as the preferred option for serine and zinc carbapenemases, respectively, if the clinical isolates were identified [20]. Although limited antibiotic options are available, optimal antibiotic regimens should be selected for each carbapenemase to maximize therapeutic outcomes and reduce mortality.

A prompt decision regarding the precise antibiotic dosing regimen is necessary to treat patients with severe infection. Integration of in vitro activity, molecular characteristics based on the geographic region and pharmacokinetic/pharmacodynamic (PK/PD) analysis using Monte Carlo simulation is typically used to design appropriate antibiotic dosing regimens to support treatment decisions. The optimal antibiotic regimen can maximize therapeutic effects, improve patient clinical outcomes and reduce patient mortality rates [21,22]. Understanding the diverse epidemiology of CRE at both the phenotype and genotype levels in each setting can contribute to making an optimal decision for treatment. However, a detailed understanding of the above aspects in Thailand remains limited. Thus, we conducted research to investigate the in vitro activity of antimicrobials against CRE and sought to identify the carbapenemase genes of clinical CRE isolates using molecular typing. Finally, we established an optimal antibiotic regimen against CRE using Monte Carlo simulation.

## 2. Results

A total of 150 nonduplicated clinical CRE isolates from various specimens were collected from 12 hospitals: 4 regional hospitals (level A) and 8 general hospitals (5 hospitals at level S and 3 hospitals at level M1). The number of CRE cases at level A (*n* = 72 of 150; 48%) was higher than that at levels S (*n* = 54 of 150; 36%) and M1 (*n* = 24 of 150; 16%).

Antimicrobial susceptibility test results were obtained for 114 strains of CRKP (76%), 33 strains of CREC (22%) and 3 strains of CREclo (2%). The total antibiotic susceptible rates were 1 (0.67%) for ertapenem, 6 (4%) for aztreonam, 17 (11.33%) for imipenem, 15 (10.00%) for meropenem, 37 (24.67%) for ceftazidime/avibactam, 85 (56.67%) for tigecycline, 103 (68.67%) for colistin, 97 (64.67%) for gentamicin, and 130 (86.67%) for amikacin. Table 1 shows the susceptibility rates, MIC 50, MIC 90 and MIC ranges for several studied antibiotics grouped by the type of CRE at each hospital level.

For antibiotic-resistant carbapenemase genes, *bla_NDM_* (*n* = 71 of 150; 47.33%), *bla_OXA-48_* (*n* = 65 of 150; 43.33%), and *bla_NDM_* plus *bla_OXA-48_* (*n* = 10 of 150; 6.67%) were detected usingmultiplex polymerase chain reaction (multiplex PCR). Of the 114 isolates of CRKP, *bla_OXA-48_* (*n* = 60 of 114; 52.63%) was the most common carbapenemase gene detected, followed by *bla_NDM_* (*n* = 43 of 114; 37.72%), *bla_NDM_* plus *bla_OXA-48_* (*n* = 9 of 114; 7.89%) and no presence of any carbapenemase gene (*n* = 2 of 114; 1.75%). According to CREC, the detected carbapenemase genes included *bla_NDM_* (*n* = 27 of 33; 81.82%), *bla_OXA-48_* (*n* = 5 of 33; 15.15%) and *bla_NDM_* plus *bla_OXA-48_* (*n* = 1 of 33; 3.03%). No carbapenemase genes (*n* = 2 of 3; 66.7%) and *bla_NDM_* (*n* = 1 of 3; 33.3%) were detected in CREclo. Furthermore, only five strains of total isolates (5 of 150, 3.3%) were positive for both *mcr-1* and *bla_OXA-48_*, while 80% (*n* = 4 of 5) of strains were colistin-resistant (colistin MIC > 8 µg/dL). Table 2 shows the type of carbapenemase genes grouped by the type of CRE at each hospital level.

Table 3 shows the percentage of susceptibility of the studied antibiotics grouped by the type of carbapenemase gene. All types of carbapenemase genes were susceptible to amikacin, whereas only *bla_NDM_* plus *bla_OXA-48_* was susceptible to gentamicin. Furthermore, the percentage of susceptibility to colistin and tigecycline was higher in *bla_NDM_*-positive isolates than in others, while the percentage of susceptibility to ceftazidime/avibactam in *bla_OXA-48_* -positive isolates was only 46.15%.

For PK/PD data, high dose antibiotic regimens of antibiotics were simulated. Most antibiotic regimens cannot achieve 90% cumulative fraction of response (CFR), except for high-dose tigecycline regimens (loading dose of 400 mg, followed by 200 mg every 12 h). Table 4 shows the CFR of several antibiotics under study.

## 3. Discussion

Currently, CRE is difficult to treat because antibiotic options remain limited. Second-line antibiotics are commonly used in clinical settings. In Thailand, the development of carbapenem resistance and the rates of NDM have increased over time; however, in vitro results are lacking concerning antimicrobial susceptibility and antibiotic-resistant genes in CRE from multi-centers in hospitals in western Thailand. This is the first study to report the susceptibility and molecular epidemiology of CRE isolates across multiple hospitals in western Thailand.

In our study, we found that three quarters (76%) of CRKP isolates were resistant, followed by CREC (22%) and CREclo (2%). Additionally, 57% of the CRKP isolates were mostly found at hospital level A. Our findings were consistent with those of Thongkoom et al., who found that the most common CRE isolates from a tertiary care hospital in Thailand were *K. pneumoniae* (290/411; 71%), followed by *E. coli* (47/411; 11.4%) and *E. cloacae* (31/411; 7.5%) [23]. In contrast, a systematic review and meta-analysis found that most CRE isolates were *K. pneumoniae* (63.6%), followed by *E. cloacae* and *E. coli* [7].

Our findings showed that 80% of all isolates in hospitals in western Thailand were susceptible to amikacin. These results agreed with related studies reporting that antimicrobial susceptibility testing of amikacin ranged from 84 to 99% [24]. Furthermore, the antibiotic resistance rates were higher in regional hospitals than in the others. The rates of resistance to colistin, tigecycline, ceftazidime/avibactam and aztreonam were higher at hospital level A than at other levels, whereas the resistance rates of aminoglycoside seldom changed. The change in the resistance rates may be caused by differences in the antibiotic use to treat patients with complicated infections at each hospital level.

The proportions of meropenem MICs for CP-CRE and non-CP-CRE at meropenem MICs ≥ 16 µg/mL were 86.3% (*n* = 126 of 146) and 50% (*n* = 2 of 4), respectively. The number of carbapenemase-producing positive isolates was higher than that of non-carbapenemase-producing isolates, which was consistent with a related study (CP-CRE vs. non-CP-CRE = 38% vs. 2% at meropenem MICs ≥ 16 µg/mL) [10]. Thus, CP-CRE may be more virulent than non-CP-CRE.

In a clinical setting, treating infections with a high dose and prolonged infusion of carbapenems or double-carbapenem regimens may be useful when KPC-positive isolates have meropenem MICs ≤ 8 µg/mL [25]. Regarding the Ambler classification, our findings revealed the most identified isolates were in Ambler classes B and D, including NDM (47.33%), OXA-48 (43.33%) and NDM plus OXA-48 (6.67%). The main carbapenemase types in our study were similar to those found in a related study that recently reported the top three carbapenemase types were NDM (46.6%), OXA-48 (25.1%) and NDM plus OXA-48 (25.1%) [16]. Nonetheless, related studies also reported other carbapenemase types, including KPC and IMP-14 [13,15]. Furthermore, MIC levels that reach the resistance level may be associated with the carbapenemase type. At meropenem MICs ≤ 8 µg/mL, the majority of the carbapenemase-positive isolates were OXA-48-producing isolates (*n* = 16 of 65; 24.62%), followed by NDM (*n* = 2 of 71; 2.82%) and NDM plus OXA-48 (*n* = 2 of 10; 20%). Similarly, in 2016, Fattouh R et al. reported that 58.93% of KPC- (*n* = 33 of 56), 40% of OXA-48 (*n* = 12 of 30) and 14.46% of NDM-positive isolates (*n* = 12 of 83) had meropenem MICs ≤ 8 µg/mL. It seemed that these NDM-producing pathogen isolates were likely to have stronger resistance levels than OXA-48- and KPC-producing isolates [11]. Overall, carbapenem-containing regimens are inappropriate for CRE treatment in our setting because none of the CRE isolates in our study had Ambler class A (KPC), and most CRE isolates provided meropenem MICs >8 µg/mL

Colistin may be one option given its activities against CRE isolates. Colistin-intermediate isolates (MIC ≤ 2 µg/mL) were found to account for 68.7% (*n* = 103 of 150) in this region. The most resistant genes associated with colistin-resistant isolates were *bla_OXA-48_* (*n* = 31 of 47; 66%) and *mcr-1* (*n* = 5 of 150; 3.3%). Interestingly, we observed the coexistence of *mcr-1* with *bla_OXA-48_* in carbapenem-resistant *K. pneumoniae.* Among these coexisting resistant genes, 80% (*n* = 4 of 5) had colistin MICs >8 µg/mL. Related studies have demonstrated that the co-occurrence of *mcr-1* and *bla_OXA-48_* is common in colistin-resistant isolates; these isolates had colistin MICs ranging from 32 to 64 µg/dL [26]. This may have been because most pathogens containing the carbapenemase and *mcr-1*-positive genes on their plasmids or integrons can carry, transfer and move genetic elements to another pathogen, leading to a high level of resistance to colistin and carbapenem [27]. For PK/PD, our study showed that none of the overall colistin regimens could achieve 90% of CFR targets; instead, they achieved 53 to 64%. The results were consistent with the study by Jitaree, reporting that the overall CFR of colistin regimens was approximately 70 to 86% [28]. Thus, colistin-containing regimens for therapy against CRE in the region should be used with caution.

The use of tigecycline in regimens may be a treatment option with higher colistin MICs or when renal failure occurs [25]. Our study showed that only 56.7% of all isolates were susceptible to tigecycline at MICs ≤ 0.5 µg/mL. These results were contrary to those of related studies; the data reported that approximately 90% of CRE remained susceptible to tigecycline [24,29]. Using a CFR > 90% for *f*AUC_0-24_/MIC ≥ 0.9, only high dose tigecycline regimens achieved the target, with a loading dose of 400 mg with a maintenance dose of 200 mg every 12 h, whereas the usual regimen (a loading dose of 200 mg with a maintenance dose of 100 mg every 12 h) achieved almost 90% CFR. The results are consistent with related studies showing that a high dose (tigecycline at 200 mg initially, followed by 100 mg every 12 h) could achieve a favorable CFR target that may lead to reduced 30-day and ICU mortality when compared with the standard dose (tigecycline 100 mg initially, followed by 50 mg every 12 h) (OR (95%CI) = 2.25 (0.55–9.24) and 12.48 (2.06–75.43), respectively) [30,31]. Therefore, high dose tigecycline regimens should be selected for serious MDR CRE infections. The safety and efficacy of various high doses of tigecycline against CRE should be investigated in large scale clinical studies.

Aztreonam and ceftazidime/avibactam are new generation antibiotics for combatting antibiotic-resistant Gram-negative bacteria. Aztreonam was not hydrolyzed by NDM, whereas ceftazidime/avibactam was not hydrolyzed by OXA-48 [32]. According to carbapenemase types in Thailand, NDM was shown to be the most common carbapenemase type [16], whereas OXA-48 was also found in some settings [17]. We hypothesized that both antibiotics may exhibit good activity against CRE. Our findings were inconsistent with the hypothesis, as 96% of all CRE isolates were resistant to aztreonam because of the background of resistance, particularly ESBLs, which have the ability to hydrolyze most penicillins, cephalosporins and monobactams (aztreonam). Ceftazidime/avibactam is highly active against class D carbapenemase; however, one half of all OXA-48-positive isolates (*n* = 35 of 65; 53.85%) showed high MIC values (≥16/4 µg/mL). These isolates develop ceftazidime/avibactam resistance, leading to increased MIC values. The resistance mechanisms may be associated with an amino acid mutation. Typically, naïve OXA-48 contained proline (Pro) at position 68 and tyrosine (Tyr) at position 211. Substitution of proline (Pro) by alanine (Ala) (Ala68Pro) at position 68 and Tyr by serine (Ser) (Ser211Tyr) at position 211 may occur in ceftazidime/avibactam-resistant isolates. Although some isolates were resistant to ceftazidime/avibactam, 25% of all isolates remained susceptible (MIC ≤ 8 µg/mL). The PK/PD profile of ceftazidime/avibactam provided a rationale for regimen optimization. Although the recommended regimens included 2.5 g every 8 h infusion 2 h, these regimens could not meet the CFR targets because of the NDM-positive isolates (*n* = 81 of 150; 54%) [20]. Overall, among the groups of new generation antibiotics, ceftazidime/avibactam may be the only option for treatment when OXA-48-positive CRE isolates are detected and are susceptible to ceftazidime/avibactam.

Antibiotic susceptibility may be determined by carbapenemase types. Our study showed that susceptibility to aminoglycosides, colistin and tigecycline differed among carbapenemase genes. The NDM-producing isolates were more susceptible to colistin and tigecycline than OXA-48- and NDM plus OXA-48-producing isolates. This finding was similar to a related study; most NDM-positive isolates remained susceptible to colistin and tigecycline [33]. Furthermore, NDM-positive isolates were resistant to aminoglycosides because they carry 16S rRNA methylase genes (*rmtF*) [34]. Our NDM-positive isolates were resistant to gentamicin, but they remained susceptible to amikacin. The findings were consistent with those of Upadhyaya P and colleagues in 2019, who reported that the loss of *rmtF* methylase genes may be associated with the loss of amikacin resistance [35]. Additionally, the antibiotic susceptibility characteristics of the OXA-48-producing isolates were similar to NDM plus OXA-48-producing isolates. They were highly resistant to all antibiotics, except amikacin. NDM plus OXA-48-producing isolates were likely to be more resistant than OXA-48-producing isolates. The number of carbapenemase genes may not be proportional to the percentage of susceptibility. Mobile genetic elements, which are related to carbapenemase gene transmission on chromosomes or plasmids, may be involved in the extensive spreading of antibiotic resistance [36]. Whole-genome sequencing should be further studied.

As mentioned above, the number of effective antibiotics against CRE remains limited. Combination antibiotic treatment with MIC-based dose optimization is a recommended strategy in the clinical setting due to improved clinical outcomes [37]. When selecting antibiotics for combination, the pathogen should be susceptible to one or both active agents, and the antibiotics should have synergistic activities for additional therapeutic effects. When combining antibiotics, the antibiotic MIC values are shifted from high to low levels (approximately a 2 fold reduction in MICs compared with MICs of single agents) [38]. In our setting, most CRE isolates were susceptible to amikacin and gentamicin; therefore, amikacin or gentamicin may have a role in antibiotic combination regimens [18]. Furthermore, in a related study in a Thai university, CRE isolates susceptible to amikacin and gentamicin (100%) showed that tigecycline plus gentamicin (13.3%) and fosfomycin plus gentamicin (30%) had synergistic activities in carbapenem- and colistin-resistant *K. pneumoniae* [39].

Several limitations were encountered in the study. First, we investigated CRE clinical isolates from hospitals in the western region, which might not be generalizable to other regions. Second, we only focused on the resistance mechanisms based on carbapenemase genes and the *mcr-1* gene. Further studies are needed to investigate the synergistic activity of combination antibiotic regimens, e.g., ceftazidime/avibactam plus aztreonam, against NDM plus OXA-48-positive isolates, non-carbapenemase resistant mechanisms, e.g., efflux pump or porin loss, or whole-genome sequencing.

## 4. Materials and Methods

### 4.1. Bacterial Strains

In this study, we collected all Enterobacterales isolates from hospitals in western Thailand. Nonetheless, only *K. pneumoniae*, *E. coli*, *E. cloacae* were resistant to carbapenems.

A total of 150 nonduplicated CRKP, CREC and CREclo isolates were obtained from the bacterial culture bank of Regional Medical Sciences Center V, Samut Songkhram, Thailand. These 150 CRE isolates were obtained from patients admitted to 12 hospitals in western Thailand from September 2019 to October 2020.

All 12 hospitals were divided in the categories of regional hospitals (level A) and general hospitals (levels S and M1). Level A hospitals are regional hospitals serving patients with more complications, with specialized staff, technical equipment and at least a 700-bed capacity. Levels S and M1 hospitals are general hospitals serving patients with complications, with specialized staff and at least a 300- and 150-bed capacity, respectively. The hospitals included in the study included four hospitals at level A, 5 hospitals at level S and 3 hospitals at level M1. A list of hospitals included in the study is presented in Supplementary Materials (Supplementary Material Table S1).

CRKP, CREC and CREclo isolates were identified by the National Institute of Health of Thailand (NIH). Carbapenem-resistant isolates were defined on the basis of their nonsusceptibility to one of the carbapenems, including ertapenem, imipenem or meropenem, according to the CLSI 2020 [40]. All isolates were stored at −80°C until analysis. *E. coli* ATCC 25922 was used as a reference strain for quality control in the study.

### 4.2. Determining MICs

A broth microdilution procedure was used to determine MICs. First, 3 to 5 colonies were picked and suspended in distilled water (4 mL), and the turbidity was adjusted to 0.5 McFarland. Next, 30 µL of the prepared bacterial suspension (~10^8^ cfu/mL) was diluted in 11 mL of cation-adjusted Mueller Hinton broth (Sensititre^TM^ cation-adjusted Mueller Hinton Broth with TES; TREK Diagnostic Systems Ltd., East Grinstead, West Sussex, UK) at ~1:1000 dilution. The final bacterial suspension contained an inoculum density of ~10^5^ cfu/mL. Then 50 µL of the final inoculum was dispensed in commercial 96-well plates (Sensititre Gram-negative DKMGN Plate, TREK Diagnostic Systems Ltd., East Grinstead, West Sussex, UK). In commercial 96-well plates, two-fold serial dilutions of meropenem, imipenem, ertapenem, amikacin, gentamicin, colistin, tigecycline, aztreonam and ceftazidime/avibactam were made, ranging from 0.12 to 16 µg/mL, 0.5 to 16 µg/mL, 0.12 to 2 µg/mL, 4 to 32 µg/mL, 0.5 to 8 µg/mL, 0.25 to 8 µg/mL, 0.25 to 4 µg/mL, 0.5 to 32 µg/mL and 0.5/4 to 16/4 µg/mL, respectively. The MICs were determined after incubating at 37 °C 18 to 20 h [41].

The MIC breakpoints were interpreted following the European on Antimicrobial Susceptibility Testing breakpoint for 2021 (EUCAST, 2021) for tigecycline [42] and the CLSI breakpoint in 2021 (CLSI, 2021) for the other antibiotics [41]. The cut-off for the intermediate MIC breakpoint was ≤2 µg/mL for colistin. Additionally, the cut-off for susceptible MIC breakpoints were ≤1 µg/mL for meropenem and imipenem, ≤0.5 µg/mL for ertapenem, ≤1 6 µg/mL for amikacin, ≤4 µg/mL for gentamicin, ≤0.5 µg/mL for tigecycline, ≤4 µg/mL for aztreonam, and ≤8/4 µg/mL for ceftazidime/avibactam. MIC 50 and MIC 90 were defined as the MICs inhibiting 50% and 90% of pathogens, respectively.

### 4.3. Molecular Study of Antibiotic Resistance Genes

A multiplex PCR technique was used to detect antibiotic resistance genes, including the most common carbapenemases (*bla_NDM_*, *bla_OXA-48_*, *bla_IMP_*, *bla_VIM_*, and *bla_KPC_*) and *mcr-1* (*mobilized colistin resistance-1*) genes. The primer set for the antibiotic resistance genes was described in a related study [43].

The multiplex PCR temperature cycle for carbapenemase genes was as follows: pre-incubation for 3 min at 94 °C, 35 cycles of 30 s at 94 °C, 35 s at 57°C, 45 s at 72 °C, and a final extension step for 5 min at 72 °C [14]. The *mcr-1* PCR protocol was as follows: pre-incubation for 5 min at 94 °C, 35 cycles of 30 s at 94 °C, 35 s at 53 °C, 45 s at 72 °C, and a final extension step for 5 min at 72 °C. Agarose (1%) gel electrophoresis in 0.5 × Tris/Borate/EDTA (TBE) stained with ethidium bromide was used to separate the DNA fragments.

### 4.4. Antibiotic Dose Optimization by Monte Carlo Simulation

The relationship between drug concentration levels and time in plasma was studied using linear one-compartment pharmacokinetic models of meropenem, gentamicin, colistin and ceftazidime/avibactam, as well as two-compartment pharmacokinetic models of imipenem, amikacin, tigecycline and colistin methanesulfonate (CMS). The pharmacokinetic parameters of critically ill patients, PK/PD indices and targets, and the antibiotic dosing regimens used in simulations are presented in Supplementary Materials (Supplementary Material Table S2) [44,45,46,47,48,49,50,51,52,53,54]. The optimal antibiotic regimens were simulated using a 10,000-subject Monte Carlo simulation (Oracle Crystal Ball Classroom Faculty Edition-Oracle 1-Click Crystal Ball 201, Thailand).

The optimal antibiotic regimens were empirically defined as target achievement above 90% CFR. The CFR is the estimated probability of each regimen calculated from the proportion of the bacterial population multiplied by probability of target attainment (PTA) at each MIC, calculated as follows: $\mathrm{CFR}=\;\sum_{i=1}^{n}{\mathrm{PTA}}_{i}\;\times F_{i}$, where the subscript i is the MIC value ranked from the lowest to highest value, PTA_i_ is the PTA of each MIC, and F_i_ is the fraction of the sample organisms in each MIC category.

### 4.5. Ethics Approval

As this study was conducted using archived bacterial isolates, the study was given an exemption from having to obtain written informed consent from the patients by the Ethics Committee for Human Research of Silpakorn University, Nakhon Pathom, Thailand (Ethics number: REC 63.0429-033-1871, issued on 13 August 2020). To ensure confidentiality of patient information, anonymous typing was used, and the data were maintained in a confidential manner.

## 5. Conclusions

CRKP, CREC and CREclo from hospitals in western Thailand were resistant to aztreonam, ceftazidime/avibactam, tigecycline and colistin, whereas they remained susceptible to amikacin and gentamicin. Regional hospitals had higher rates of resistance than general hospitals. The most common mechanisms of carbapenem resistance were NDM and OXA-48 enzymes. All isolates carrying the *mcr-1* gene also carried the *bla_OXA-48_* gene. Combination regimens with high-dose tigecycline should be considered as an optimal regimen for empirical therapy against CRE isolates.

## Figures and Tables

**Table 1:** Susceptible rates, MIC 50, MIC 90, and MIC ranges of *Klebsiella pneumoniae*, *Escherichia coli* and *Enterobacter cloacae* for several studied antibiotics grouped by the type of CRE at each hospital level.

Type of CRE	Hospital Levels	Total Isolates	MEM	IMP	ERT	AMK	GEN	COL ^$^	TGC	CZA	ATM
*Klebsiella pneumoniae*	A	65	10 (15.38%)	11 (16.92%)	0 (0%)	52 (80%)	44 (67.69%)	39 (60%)	31 (47.69%)	26 (40%)	2 (3.08%)
	S	37	1 (2.7%)	1 (2.7%)	0 (0%)	36 (97.3%)	34 (91.89%)	22 (59.46%)	16 (43.24%)	4 (10.81%)	0 (0%)
	M1	12	0 (0%)	0 (0%)	0 (0%)	11 (91.67%)	10 (83.33%)	10 (83.33%)	6 (50%)	2 (16.67%)	0 (0%)
	Total	114	11 (9.65%)	12 (10.53%)	0 (0%)	99 (86.85%)	88 (77.2%)	71 (62.29%)	53 (46.5%)	32 (28.08%)	2 (1.76%)
	MIC 50		>16	16	>2	8	1	1	1	>16/4	>32
	MIC 90		>16	>16	>2	>32	>8	>8	2	>16/4	>32
	MIC range		0.5–>16	≤0.5–>16	NR	≤4–>32	≤0.5–>8	0.5–>8	≤0.25–>4	≤0.5/4–>16/4	≤0.5–>32
*Escherichia coli*	A	7	0 (0%)	1 (14.29%)	0 (0%)	6 (85.71%)	2 (28.57%)	7 (100%)	6 (85.71%)	0 (0%)	2 (28.57%)
	S	15	1 (6.67%)	1 (6.67%)	0 (0%)	15 (100%)	3 (20%)	14 (93.33%)	14 (93.33%)	1 (6.67%)	1 (6.67%)
	M1	11	2 (18.18%)	1 (9.09%)	1 (9.09%)	8 (72.73%)	3 (27.27%)	8 (72.73%)	11 (100%)	2 (18.18%)	1 (9.09%)
	Total	33	3 (9.09%)	3 (9.09%)	1 (3.03%)	29 (87.88%)	8 (24.24%)	29 (87.88%)	31 (93.94%)	3 (9.09%)	4 (12.12%)
	MIC 50		>16	16	>2	≤4	>8	1	≤0.25	>16/4	>32
	MIC 90		>16	>16	>2	32	>8	4	0.5	>16/4	>32
	MIC range		≤0.125–>16	≤0.5–>16	≤0.125–>2	≤4–>32	≤0.5–>8	1–8	≤0.25–1	≤0.5/4–>16/4	2–>32
*Enterobacter cloacae*	A	0	NR	NR	NR	NR	NR	NR	NR	NR	NR
	S	2	1 (50%)	1 (50%)	0 (0%)	2 (100%)	1 (50%)	2 (100%)	1 (50%)	1 (50%)	0 (0%)
	M1	1	0 (0%)	1 (100%)	0 (0%)	0 (0%)	0 (0%)	1 (100%)	0 (0%)	1 (100%)	0 (0%)
	Total	3	1 (33.33%)	2 (66.67%)	0 (0%)	2 (66.67%)	1 (33.33%)	3 (100%)	1 (33.33%)	2 (66.67%)	0 (0%)
	MIC 50		>16	≤0.5	>2	≤4	>8	1	1	1/4	>32
	MIC 90		>16	>16	>2	>32	>8	1	1	>16/4	>32
	MIC range		≤0.125–>16	≤0.5–>16	2–>2	≤4–>32	≤0.5–>8	NR	0.5–1	1/4–>16/4	NR
Total	A	72	10 (13.89%)	12 (16.67%)	0 (0%)	58 (80.56%)	46 (63.89%)	46 (63.89%)	37 (51.39%)	26 (36.11%)	4 (5.56%)
	S	54	3 (5.56%)	3 (5.56%)	0 (0%)	53 (98.15%)	38 (70.37%)	38 (70.37%)	31 (57.41%)	6 (11.11%)	1 (1.85%)
	M1	24	2 (8.33%)	2 (8.33%)	1 (4.17%)	19 (79.17%)	13 (54.17%)	19 (79.17%)	17 (70.83%)	5 (20.83%)	1 (4.17%)
	Total	150	15 (10%)	17 (11.33%)	1 (0.67%)	130 (86.67%)	97 (64.67%)	103 (68.67%)	85 (56.67%)	37 (24.67%)	6 (4%)
	MIC 50		>16	16	>2	8	1	0.5	0.5	>16/4	>32
	MIC 90		>16	>16	>2	>32	>8	>8	2	>16/4	>32
	MIC range		≤0.125–>16	≤0.5–>16	≤0.125–>2	≤4–>32	≤0.5–>8	0.5–>8	≤0.25–>4	≤0.5/4–>16/4	≤0.5–>32

Abbreviations: MEM: meropenem; IMP: imipenem; ERT: ertapenem; AMK: amikacin; GEN: gentamicin; COL: colistin; TGC: tigecycline; CZA: ceftazidime/avibactam; ATM: aztreonam; NR: not reported; CRE: carbapenem-resistant Enterobacterales; ^$^ using the intermediate breakpoint following the Clinical & Laboratory Standards Institute (CLSI) 2021.

**Table 2:** Type of carbapenemase genes grouped by the type of CRE at each hospital level.

Type of CRE	Hospital Levels	Total Number of Isolates	*bla_NDM_*	*bla_OXA-48_*	*bla_NDM_* Plus *bla_OXA-48_*	No Carbapenemase Genes	*mcr-1*
*Klebsiella pneumoniae*	A	65	20 (30.77%)	40 (61.54%)	5 (7.69%)	0 (0%)	5 (7.69%)
	S	37	18 (48.65%)	14 (37.84%)	4 (10.81%)	1 (2.7%)	0 (0%)
	M1	12	5 (41.67%)	6 (50%)	0 (0%)	1 (8.33%)	0 (0%)
	Total	114	43 (37.72%)	60 (52.63%)	9 (7.89%)	2 (1.75%)	0 (0%)
*Escherichia coli*	A	7	6 (85.71%)	1 (14.29%)	0 (0%)	0 (0%)	0 (0%)
	S	15	12 (80%)	2 (13.33%)	1 (6.67%)	0 (0%)	0 (0%)
	M1	11	9 (81.82%)	2 (18.18%)	0 (0%)	0 (0%)	0 (0%)
	Total	33	27 (81.82%)	5 (15.15%)	1 (3.03%)	0 (0%)	0 (0%)
*Enterobacter* *cloacae*	A	NR	NR	NR	NR	NR	NR
	S	2	1 (50%)	0 (0%)	0 (0%)	1 (50%)	0 (0%)
	M1	1	0 (0%)	0 (0%)	0 (0%)	1 (100%)	0 (0%)
	Total	3	1 (33.33%)	0 (0%)	0 (0%)	2 (66.67%)	0 (0%)
Total	A	72	26 (36.11%)	41 (56.94%)	5 (6.94%)	0 (0%)	5 (6.94%)
	S	54	31 (57.41%)	16 (29.63%)	5 (9.26%)	2 (3.7%)	0 (0%)
	M1	24	14 (58.33%)	8 (33.33%)	0 (0%)	2 (8.33%)	0 (0%)
	Total	150	71 (47.33%)	65 (43.33%)	10 (6.67%)	4 (2.67%)	5 (3.33%)

Abbreviations: NDM: New Delhi metallo-β-lactamase; OXA-48: oxacillinase-48, *mcr-1*: mobilized colistin resistance-1; NR: not reported; CRE: carbapenem-resistant Enterobacterales.

**Table 3:** Susceptible rates, MIC range, MIC 50 and MIC 90 of all antibiotics grouped by type of carbapenemase gene.

Carbapenemase Genes	*bla_NDM_* (*n* = 71)				*bla_OXA-48_* (*n* = 65)				*bla_NDM_* Plus *bla_OXA-48_* (*n* = 10)			
Antibiotics	MIC Range	MIC 50	MIC 90	%S	MIC Range	MIC 50	MIC 90	%S	MIC Range	MIC 50	MIC 90	%S
MEM	1–>16	>16	>16	2.82	≤0.125–>16	>16	>16	16.92	1–>16	>16	>16	10.00
IMP	≤0.5–>16	16	>16	2.82	≤0.5–>16	16	>16	16.92	≤0.5–>16	>16	>16	10.00
ERT	2–>2	>2	>2	0	≤0.125–>2	>2	>2	1.54	>2	>2	>2	0
AMK	≤4–>32	≤4	32	88.73	≤4–>32	8	>32	86.15	≤4–>32	8	8	80.00
GEN	≤0.5–>8	2	>8	54.93	≤0.5–>8	2	>8	73.85	≤0.5–>8	1	2	80.00
COL ^$^	1–>8	1	8	85.92	≤0.5–>8	8	16	52.31	1–>8	4	>8	40.00
TGC	≤0.25–>4	0.5	1	73.24	≤0.25–>4	1	2	43.08	≤0.25–2	1	1	40.00
CZA	≤0.5/4–>16/4	>16/4	>16/4	2.82	≤0.5/4–>16/4	>16/4	>16/4	46.15	1/4–>16/4	>16/4	>16/4	20.00
ATM	≤0.5–>32	>32	>32	7.04	4–>32	>32	>32	1.54	>32	>32	>32	0

Abbreviations: NDM: New Delhi metallo-β-lactamase; OXA-48: oxacillinase-48; MEM: meropenem; IMP: imipenem; ERT: ertapenem; AMK: amikacin; GEN: gentamicin; COL: colistin; TGC: tigecycline; CZA: ceftazidime/avibactam; ATM: aztreonam. $ using the intermediate breakpoint following the Clinical & Laboratory Standards Institute (CLSI) 2021.

**Table 4:** Cumulative fraction of response (CFR) of several studied antibiotics grouped by the type of carbapenem-resistant Enterobacterales (CRE).

Antibiotics	LD	MD	Infusion Time	% CFR			
				*K. pneumoniae*	*E. coli*	*E. cloacae*	All
MEM	2 g	1 g inf q 8 h	0.5 h	16.78	14.45	34.40	16.62
	2 g	1 g inf q 6 h	0.5 h	25.66	23.20	41.26	25.43
	2 g	1 g inf q 8 h	3 h	20.30	17.60	37.23	20.04
	2 g	1 g inf q 6 h	3 h	32.00	29.48	45.72	31.72
IMP	1 g	0.5 g inf q 6 h	2 h	7.36	5.39	48.47	7.75
	1 g	0.5 g inf q 6 h	3 h	9.33	7.07	56.86	9.77
	1 g	1 g inf q 6 h	2 h	11.25	8.68	59.01	11.63
	1 g	1 g inf q 6 h	3 h	13.30	10.37	63.83	13.66
AMK	25 mg/kg	15 mg/kg q 24 h	0.5 h	21.63	39.93	38.18	25.98
	25 mg/kg	20 mg/kg q 24 h	0.5 h	28.05	51.59	49.25	33.65
	30 mg/kg	15 mg/kg q 24 h	0.5 h	22.75	41.96	40.10	27.32
	30 mg/kg	20 mg/kg q 24 h	0.5 h	28.58	52.51	50.11	34.27
GEN	7 mg/kg	5 mg/kg q 24 h	0.5 h	60.71	15.73	33.33	50.27
	7 mg/kg	6 mg/kg q 24 h	0.5 h	60.68	15.72	33.33	50.24
	8 mg/kg	5 mg/kg q 24 h	0.5 h	60.79	15.77	33.32	50.34
	8 mg/kg	6 mg/kg q 24 h	0.5 h	63.58	16.99	33.33	52.73
	8 mg/kg	7 mg/kg q 24 h	0.5 h	65.32	17.82	33.33	54.23
COL	300 mg	150 mg q 12 h	0.5 h	48.28	66.42	74.75	52.81
	300 mg	150 mg q 8 h	0.5 h	59.06	77.44	84.20	63.61
	300 mg	180 mg q 12 h	0.5 h	48.20	66.21	74.60	52.69
	300 mg	180 mg q 8 h	0.5 h	58.95	77.81	84.75	63.62
TGC	200 mg	100 mg q 12 h	0.5 h	84.31	100.00	100.00	88.07
	200 mg	100 mg q 24 h	0.5 h	62.27	97.24	69.67	70.11
	400 mg	100 mg q 12 h	0.5 h	84.67	100.00	100.00	88.34
	400 mg	100 mg q 24 h	0.5 h	62.35	97.26	69.86	70.18
	400 mg	200 mg q 12 h	0.5 h	94.00	100.00	100.00	95.44
	400 mg	200 mg q 24 h	0.5 h	84.59	100.00	100.00	88.28
CZA	-	2.5 g q 8 h	0.5 h	72.76	40.79	77.00	50.45
	-	2.5 g q 8 h	1 h	73.50	41.70	77.34	51.22
	-	2.5 g q 8 h	2 h	76.66	44.45	79.00	53.74
	-	2.5 g q 8 h	3 h	78.94	48.15	80.34	56.86

Abbreviations: MEM: meropenem; IMP: imipenem; AMK: amikacin; GEN: gentamicin; COL: colistin; TGC: tigecycline; CZA: ceftazidime/avibactam; loading dose; MD: maintenance dose.

## Data Availability

Data are available on reasonable request due to restrictions.

## Supplementary Materials

The following supporting information can be downloaded at: https://www.mdpi.com/article/10.3390/antibiotics11030355/s1, Table S1: List of hospitals in the study. Table S2: Pharmacokinetic parameters of critically ill patients.
