# Supplementary material for: Antimicrobial Activity Profiles and Potential Antimicrobial Regimens against Carbapenem-Resistant Enterobacterales Isolated from Multi-Centers in Western Thailand
Source: Antibiotics (Basel). 2022 Mar 7;11(3):355. doi: 10.3390/antibiotics11030355 (PMC8944502; doi:10.3390/antibiotics11030355)
Supplement: Supplementary file 1 [file antibiotics-11-00355-s001.zip › antibiotics-1602058-supplementary.pdf]

antibiotics

Supplementary Materials

Table S1. List of hospitals in the study.

Hospitals levels	Hospital Names	Provinces
A	Chaophraya Yommaraj Hospital	Suphan Buri
	Nakhon Pathom Hospital	Nakhon Pathom
	Ratchaburi Hospital	Ratchaburi
	Samut Sakhon Hospital	Samut Sakhon
S	Banphaeo General Hospital [BGH]	Samutsakhon
	Prachuapkhirikhan Hospital	Prachuap Khiri Khan
	King Mongkut Memorial Hospital	Phetchaburi
	Somdej Phra Phutthaloetla Hospital	Samut Songkhram
	Hua Hin Hospital	Prachuap Khiri Khan
M1	Photharam Hospital	Ratchaburi
	Krathumbaen Hospital	Samut Sakhon
	Somdejprasangkharach XVII Hospital	Suphan Buri

Abbreviations: A: advance-level hospitals; S: standard-level hospitals; M1: middle-level hospitals

Table S2. Pharmacokinetic parameters of critically ill patients.

Antibiotics	Cpt	Pharmacokinetic Parameters	PK/PD Index and Target	Antibiotic Dosing Regimens
Meropenem [44]	1	Estimate (% RSE): CL (L/h) = 7.82 (22.1), V (L) = 23.7 (12.6)	100% f T > MIC [45]	Loading dose 2 g, followed by 1 g infusion 0.5–3 h every 6–8 h
Imipenem [46]	2	Mean (SD): CL (L/h) = 20.86 (6.130), V (L) = 23.59 (5.070), K_{12} (/h) = 2.819 (1.448), K_{21}(/h) = 5.598 (5.283), K_e (/h) = 0.901 (0.283)	100% f T > MIC [45]	Loading dose 1 g, followed by 0.5–1 g infusion 2–3 h every 6 h
Amikacin [47]	2	Estimate (% RSE): CL (L/h) = 0.77 (28.4), V1 (L) = 19.2 (5.31) Q (L/h) = 4.38 (18.3), V2 (L) = 9.38 (7.15)	fC_{max}: MIC at least 8–10 [49]	Loading dose 25–30 mg/kg, followed by 15–20 mg/kg q 24 h
Gentamicin [48]	1	Estimate (% IIV): CL (L/h) = 3.14 (83.7), V (L) = 53.0 (64.4)	fC_{max}: MIC at least 8–10 [49]	Loading dose 7–8 mg/kg, followed by 5–7 mg/kg q 24 h
Tigecycline [50]	2	Estimate (% RSE): CL (L/h) = 22.1 (3.16), V1 (L) = 162 (5.3) Q (L/h) = 69.4 (32.6), V2 (L) = 87.9 (8.67)	$fAUC_{0-24}$/MIC ≥ 0.9 [51]	Loading dose 200–400 mg, followed by 100–200 mg every 12 h or 100–200 mg every 24 h
CMS/ Colistin [52]	2/1	Estimate (% IIV): CMS: CLD1 (L/h) = 9.57 (80.1), CLR_{SLOPE} (L/h/CrCL) = 0.0340 (75.2),	$fAUC_{0-24}$/MIC ≥ 25 [53]	Colistin: loading dose 300 mg, followed by 150-180 mg every 8-12 h in patients with creatinine clearance 91-130 ml/min

Antibiotics	Cpt	Pharmacokinetic Parameters	PK/PD Index and Target	Antibiotic Dosing Regimens
		$CLNR_{CMS}$ (L/h) = 2.52 (39.8), V1 (L) = 12.9 (40.4), V2 (L) = 16.1 (70.9) <u>Colistin:</u> CLT_c/fm (L/h) = 3.59 (37.9), V3/fm (L) = 57.2 (43.5)		
Ceftazidime/ Avibactam [54]	1	Mean (SD): <u>Ceftazidime:</u> CL (L/h) = 6.14 (3.80), V (L) = 34.78 (10.49) <u>Avibactam:</u> CL (L/h) = 11.09 (6.78), V (L) = 50.81 (14.32)	- Ceftazidime: 100% *f* T > MIC, - Avibactam: 100%*f*T > 1 µg/ml*	2.5 g every 6 or 8 h

Abbreviations: $fAUC_{0-24}$: Free drug area under the curve 0–24 h, %*f* T, > MIC: The percentage of free drug time exceeding the MIC, MIC: Minimum inhibitory concentration, fC_{max}: Free maximum drug concentration, Cpt: Compartment model, V1: Central volume of distribution, V2: Peripheral volume of distribution, Q: Intercompartmental clearance, CL: Total body clearance, k_{12}: Intercompartmental transfer rate constant from the central to peripheral compartment; k_{21}: Intercompartmental transfer rate constant from the peripheral to central compartment; k_e: Elimination rate constant from the central compartment, CMS: Colistin methanesulfonate; CLD1: Distributional clearance between the central and peripheral compartments for CMS, CLR_{SLOPE}: Slope of the relationship between renal clearance of CMS and creatinine clearance, $CLNR_{CMS}$: Non-renal clearance of CMS, V3/fm: Volume of distribution of formed colistin (V3) conditioned on the unknown fraction (fm) of the non-renal clearance of CMS that forms colistin, CLTc/fm: Clearance of colistin (CLTc) conditioned on fm; *For avibactam: a free concentration above 1 µg/mL (*f*T > 1 µg/mL) for 100% of the dosing interval.

Reference

44. Jaruratanasirikul, S.; Thengyai, S.; Wongpoowarak, W.; Wattanavijitkul, T.; Tangkitwanitjaroen, K.; Sukarnjanaset, W.; Jullangkoon, M.; Samaeng, M. Population pharmacokinetics and Monte Carlo dosing simulations of meropenem during the early phase of severe sepsis and septic shock in critically ill patients in intensive care units. *Antimicrob Agents Chemother* **2015**, *59*, 2995-3001.
45. Guilhaumou, R.; Benaboud, S.; Bennis, Y.; Dahyot-Fizelier, C.; Dailly, E.; Gandia, P.; Goutelle, S.; Lefeuvre, S.; Mongardon, N.; Roger, C.; et al. Optimization of the treatment with beta-lactam antibiotics in critically ill patients—guidelines from the French Society of Pharmacology and Therapeutics (Société Française de Pharmacologie et Thérapeutique—SFPT) and the French Society of Anaesthesia and Intensive Care Medicine (Société Française d'Anesthésie et Réanimation—SFAR). *Crit. Care* **2019**, *23*, 104.
46. Jaruratanasirikul, S.; Aeinlang, N.; Jullangkoon, M.; Wongpoowarak, W. Pharmacodynamics of imipenem in critically ill patients with ventilator-associated pneumonia. *J Med Assoc Thai* **2013**, *96*, 551-557.
47. Delattre, I.K.; Musuamba, F.T.; Nyberg, J.; Taccone, F.S.; Laterre, P.F.; Verbeeck, R.K.; Jacobs, F.; Wallemacq, P.E. Population pharmacokinetic modeling and optimal sampling strategy for Bayesian estimation of amikacin exposure in critically ill septic patients. *Ther Drug Monit* **2010**, *32*, 749-756.
49. Craig WA. Optimizing aminoglycoside use. *Crit Care Clin* **2011**, *27*, 107-121.
48 Rea, R.S.; Capitano, B.; Bies, R.; Bigos, K.L.; Smith, R.; Lee, H. Suboptimal aminoglycoside dosing in critically ill patients. *Ther Drug Monit* **2008**, *30*, 674-681.
50. Borsuk-De Moor, A.; Rypulak, E.; Potrec, B.; Piwowarczyk, P.; Borys, M.; Sysiak, J.; Onichimowski, D.; Raszewski, G.; Czuczwar, M.; Wiczling, P. Population Pharmacokinetics of High-Dose Tigecycline in Patients with Sepsis or Septic Shock. *Antimicrob Agents Chemother* **2018**, *62*, 11.
51. Bhavnani, S.M.; Rubino, C.M.; Hammel, J.P.; Forrest, A.; Dartois, N.; Cooper, C.A.; Korth-Bradley, J.; Ambrose, P.G. Pharmacological and patient-specific response determinants in patients with hospital-acquired pneumonia treated with tigecycline. *Antimicrob Agents Chemother* **2012**, *56*, 1065-1072.
52. Nation, R.L.; Garonzik, S.M.; Thamlikitkul, V.; Giamarellos-Bourboulis, E.J.; Forrest, A.; Paterson, D.L.; Li, J.; Silveira, F.P. Dosing guidance for intravenous colistin in critically-ill patients. *Clin. Infect. Dis.* **2017**, *64*, 565-571.
53. Tsala, M.; Vourli, S.; Georgiou, P.-C.; Pournaras, S.; Tsakris, A.; Daikos, G.L.; Mouton, J.W.; Meletiadis, J. Exploring colistin pharmacodynamics against *Klebsiella pneumoniae*: a need to revise current susceptibility breakpoints. *J. Antimicrob. Chemother.* **2018**, *73*, 953-961.

54. Stein, G.E.; Smith, C.L.; Scharmen, A.; Kidd, J.M.; Cooper, C.; Kuti, J.; Mitra, S.; Nicolau, D.P.; Havlichek, D.H. Pharmacokinetic and Pharmacodynamic Analysis of Ceftazidime/Avibactam in Critically Ill Patients. *Surg Infect (Larchmt)* **2019**, *20*, 55-61.
